# Nottingham Children's Hospital

**Published:** 1899-02-25

**Authors:** 


### EDITOR'S LETTER-BOX.

[Our Correspondents are reminded that prolixity is a great bar to publication, and that brevity of style and conciseness of statement greatly facilitate early insertion.]

### NOTTINGHAM CHILDREN'S HOSPITAL.

Miss C. A. Morrish writes: In your last issue "Sister Lettice" misquotes me as to the enemata, and then complains of my "inaccuracy." I adhere to my former statement, and have nothing more to say on the subject. "Sister Lettice" also adds that she found it quite possible to live a comfortable, clean, and r fined life in the isolation ward. This may be so, but there are different ideas of what is cleanly and refined. I imagine that most gentlefolk would draw the line at only a doctor's basin or a sink to wash in.

*** We think this correspondence had better cease unless some authoritative explanation or official denial can be given in respect to the charges formulated by Miss Morrish. We shall, however, be glad to insert anything truly germane to the question.—Ed. *T.H.*

